# Annenberg Hotkeys: A Free, Simple, Interactive Learning Platform

**DOI:** 10.7759/cureus.81412

**Published:** 2025-03-29

**Authors:** Elizabeth Sanseau, Kyle Cassidy, Vanessa Denny, Ebor Jacob James, Vinay Nadkarni

**Affiliations:** 1 Department of Emergency Medicine, Children's Hospital of Philadelphia, Philadelphia, USA; 2 Annenberg Extended Reality Lab, Annenberg School for Communication, University of Pennsylvania, Philadelphia, USA; 3 Department of Anesthesiology and Critical Care Medicine, Children's Hospital of Philadelphia, Philadelphia, USA; 4 Division of Pediatric Critical Care Medicine, Children's Hospital of Philadelphia, Philadelphia, USA; 5 Department of Pediatric Critical Care, Christian Medical College, Vellore, Vellore, IND

**Keywords:** free and openly accessible simulation-based case creation, in person and distance learning, low-cost simulation module creation, video augmented simulation education, video creation technical report

## Abstract

This technical report outlines the development and implementation of Annenberg Hotkeys, a free, accessible platform designed to help educators create interactive, synchronous learning modules for both in-person and remote teaching. Annenberg Hotkeys was developed during the SARS-CoV-2 pandemic to address the urgent need for innovative distance learning methods. Annenberg Hotkeys enables users to create interactive, ‘choose your own adventure’-style modules that offer multiple paths and outcomes based on learner decisions. This report outlines the complete process of developing a video-enhanced simulated scenario designed for healthcare professional education, providing practical insights for educators. The authors outline their process of creating the simulation scenario flowchart, making professional-quality videos, linking the videos to computer keyboard hotkeys, and delivering the distance medical education simulation learning activity. While pre-made modules are available online, Annenberg Hotkeys also allows educators to develop customized content tailored to their unique learning objectives. The educational goal of this tool is to improve engagement between learners and facilitators both in person and at distance.

## Introduction

Simulation-based education is a widely used instructional approach in medical training that utilizes controlled, simulated clinical environments, ranging from high-fidelity manikins to virtual, augmented, or video-based training modules, to enhance learning, critical thinking, and clinical decision-making in a safe and structured manner. The COVID-19 pandemic forced a rapid transition from traditional in-person training to virtual or computer-based simulation modalities. Educators and technologists at the University of Pennsylvania responded by developing innovative approaches to deliver equitable, experiential learning using both existing and newly created digital platforms.

The Annenberg School for Communication at the University of Pennsylvania partnered with physicians at the Children’s Hospital of Philadelphia (CHOP) to develop and test distance learning techniques using filmmaking and the free, openly accessible software Annenberg Hotkeys [1]. Annenberg Hotkeys is a user-friendly learning platform that enables facilitators to incorporate interactive videos into training modules, providing a flexible and scalable approach to simulation-based education. Designed in the spring of 2020 at the height of the COVID-19 lockdown, Annenberg Hotkeys was initially developed to train medical students in COVID-19 diagnosis and treatment when traditional bedside instruction was unavailable. It was rapidly deployed to support remote clinical training at Penn Medicine and CHOP, ensuring continuity of medical education during pandemic-related restrictions.

Beyond its initial application, Annenberg Hotkeys has been adapted for a variety of simulation-based training scenarios, including pediatric emergency medicine telesimulation in low-resource settings. By enabling instructors to create interactive, “choose your own adventure” video training modules, Annenberg Hotkeys enhances remote learning for healthcare providers in traditionally underserved areas. The software is compatible with web browsers such as Google Chrome© (Google, Mountain View), Opera© (Opera, Oslo) or Microsoft Edge© (Microsoft Corporation, Redmond) and can be used in hybrid (in-person or remote) learning environments. Additionally, Annenberg Hotkeys does not require continuous internet connectivity facilitators and learners can download modules ahead of time, making it particularly valuable in settings with limited digital infrastructure.

One of the key advantages of Annenberg Hotkeys is that facilitators can run simulation modules remotely, increasing access to high-quality training without requiring travel or extensive resource investment. This aligns with the broader goal of democratizing education, which promotes equitable access to learning opportunities regardless of socioeconomic, geographic, or demographic constraints [2]. Annenberg Hotkeys is one tool to engage in hybrid (either in person or remote) learning in a synchronous fashion. “Synchronous” education is a mode of learning in which instructors and students interact in real time through scheduled classes, video conferencing, or live chat, fostering immediate engagement and collaboration [3]. The Healthcare Simulation Dictionary (2.1) defines Remote Simulation as a modality in which facilitators, learners, or both are in separate locations but engage in real-time or asynchronous training via web conferencing tools. Mixed Reality Human Simulation, another application of Annenberg Hotkeys, involves integrating video, augmented reality, or virtual reality (VR) with physical manikins to create immersive training experiences [4].

Annenberg Hotkeys has been effectively implemented in both remote and mixed-reality human simulation, demonstrating feasibility and improvements in process-of-care outcomes, time-critical interventions, and leadership skills in both simulated and real clinical settings [5]. Co-authors (EJJ and VD) are currently using the Hotkeys method to train healthcare providers in low- and middle-income countries (LMICs), such as India and Ghana. By leveraging locally produced videos featuring real patients and clinical scenarios, they are contextualizing training materials to reflect the specific needs of healthcare workers in these regions.

The impact of Annenberg Hotkeys has been recognized in academic forums, with prior presentations at international conferences, including IPSSW2023 in Lisbon, Portugal (Sanseau E, Cassidy K. Annenberg Hotkeys Interactive Distance Learning Modules with a Virtual Reality Component. IPSSW2023 Lisbon Portugal; May 19, 2023), IPSSV2020 (Jacob James E, Vyasam S, Sanseau E, Cassidy K, Ramachandra G, Nadkarni V. Impact of Telesimulation Training on Recognition and First Hour Management of Pediatric Shock in the ER. IPSSV2020: A Virtual Experience; September 2021), SIMULUS 6 (Jacob James E, Vyasam S, Sanseau E, Cassidy K, Ramachandra G, Nadkarni V. Feasibility and Perceived Efficacy of a Novel Telesimulation Method for Training Pediatric Emergency Medicine Teams in Vellore, India. SIMULUS 6 - 6th Annual National Conference of PediSTARS in Healthcare Simulation, Virtual; April 2021), and the Weill Cornell International Telesimulation Conference (Jacob James E, Vyasam S, Sanseau E, Cassidy K, Ramachandra G, Nadkarni V. Impact of Telesimulation Training on Recognition and First Hour Management of Pediatric Shock in the ER. Building a sustainable future: 2nd Annual International Telesimulation in Healthcare conference. Weill Cornell Medicine New York Presbyterian Simulation Center, Virtual; September 2021). Its continued adaptation and evaluation in global medical education further highlight its potential to enhance simulation-based learning worldwide.

## Technical report

Creating a module

The first step in creating an effective Annenberg Hotkeys module is to develop a detailed medical scenario that outlines key clinical events, decision points, and learning objectives. Educators can create a custom simulation scenario or adapt free, pre-existing ones from resources like the Emergency SimBox [6] or the Virtual Resuscitation Room [7], which offer structured clinical scenarios for educational use.

Defining Key Decision Points and Clinical Flow

Outline the key decision points and actions learners are expected to take when evaluating and managing the patient’s condition. For example, identify points where learners might assess vital signs, administer oxygen, or initiate advanced interventions. When contextualizing the platform for a specific environment, we suggest using patient videos and resources from that specific environment to make the simulated activity feel more real to the learner. Users should address ethical considerations of both the patient and caregiver as well as the healthcare institutions when taking real patient videos, including implementing formal consent processes.

**Figure 1:**
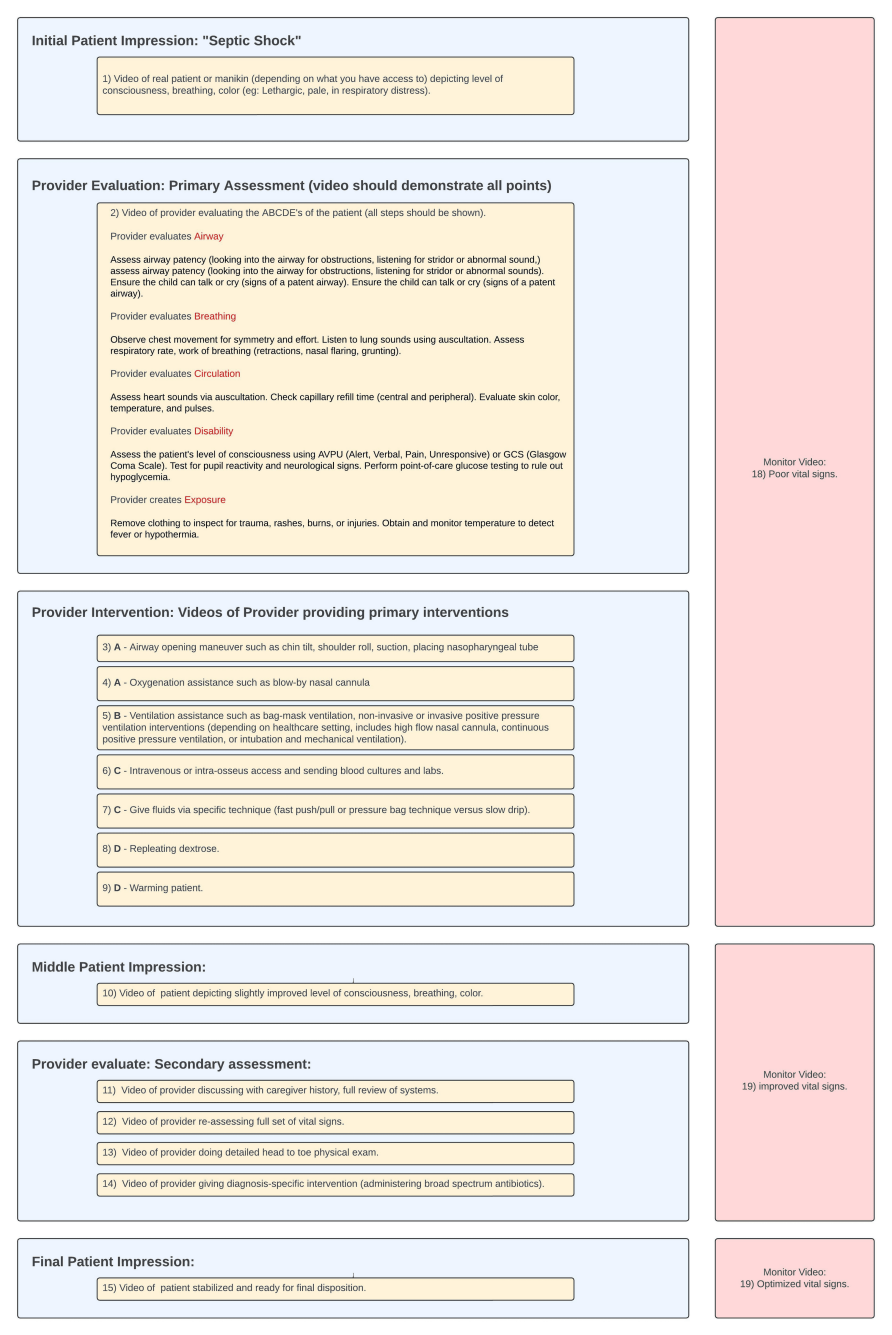
Suggested videos to create for a pediatric septic shock scenario

Videos can be obtained through the different stages of the illness -- initial presentation, deterioration, and recovery -- and then incorporated into hotkeys for the training module. In tandem, create several sets of vital signs to reflect an evolving state: poor, improving, and improved vitals. For example, in a pediatric septic shock scenario, poor vitals might align with the patient’s initial presentation, improving vitals might appear alongside airway maneuvers or oxygen administration, and fully improved vitals after fluid resuscitation and antibiotics. Figure 1 depicts an outline of 15 possible video clips to either take yourself in your unique healthcare setting for use with the Annenberg Hotkeys software, or simply download from what is already made on the website [1].

**Figure 2:**
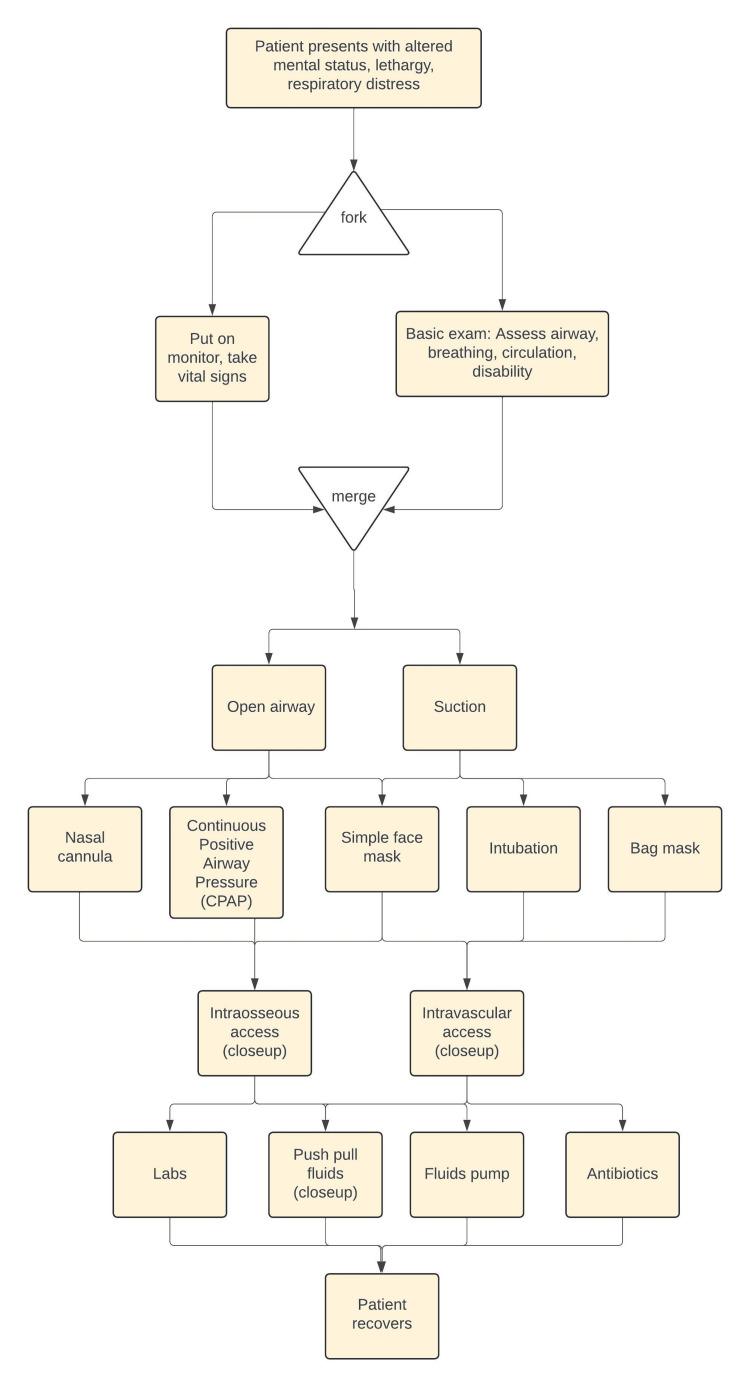
Flowchart of anticipated interventions healthcare providers would do to recognize and treat a pediatric patient with sepsis

­­Another approach is to map out the case progression using a flowchart of anticipated interventions. While a logical flow can be depicted (Figure 2), Annenberg Hotkeys’ random-access nature allows videos to be played in any order, enabling flexible and interactive learning.

Video Production and Integration of Monitors

**Figure 3:**
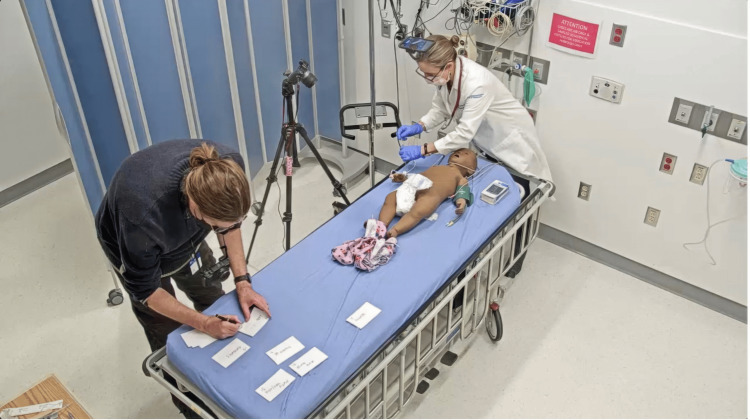
Setup of filming videos Note the camera on the tripod and the phone camera on gooseneck (above patient) as well as notecards being used to differentiate scenes and keep track of what has been filmed.

For videos hosted on the Annenberg Hotkeys website, we coordinated with a simulation technician to access a simulated hospital room equipped with a manikin, medical equipment, and supplies. The technician ran the simulation case using B-Line Medical© (Laerdal Medical, Norway) to manage the patient’s vital sign monitor and simulate patient responses [8]. Filming was completed in a one-hour session. To stay organized, we placed labeled flashcards with scene names at the foot of the bed (Figure 3).

For those with access to a simulation room, we recommend recording everything in a single take and synchronizing the footage in a video editing application like Adobe Premiere© (Adobe Inc., California). This ensures the correct monitor display aligns with each scene with minimal synchronization effort.

There are several ways that monitors can be added to a simulation. The best way to add monitors to your video clips is to record them at the same time in your sim center with the monitor software controlled in real time by a skilled operator. During the shooting process, record a single long take on all cameras simultaneously, capturing the sim center’s monitor output as a separate video feed. Use verbal cues and visual markers in between video clips. For example, say “Scene two, adding monitors” while also holding up a card with the scene name written on it. In post-production, import all video tracks into your editing software, such as Adobe Premiere©. Create a separate sequence for each camera angle and the output. Use the clapperboard or sync point to align all video tracks, and verify synchronization by checking audio waveforms or visual cues across tracks. To integrate the monitor content, place the sim center’s monitor output on the top video layer. Create a mask around the monitor area, removing all other content from this layer. Adjust the masked monitor layer’s position to align with monitors in other camera angles. When editing, cut between different camera angles as desired in your main sequence, ensuring the masked monitor layer remains on top throughout the edit. Fine-tune the monitor position for each shot if necessary. Finally, export your video with the integrated monitor overlay.

**Figure 4:**
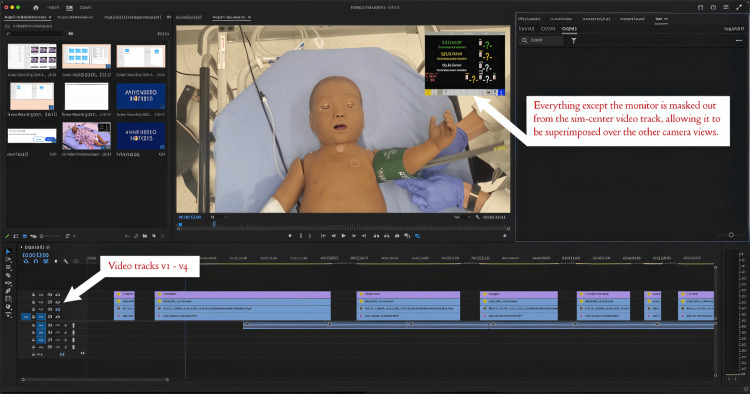
Screenshot of editing a video in Adobe Premiere

In this Adobe Premiere editing layout for synchronized multi-camera footage, the top video tracks contain the sim center videos with the monitor overlay. Below these are the overhead and side camera tracks captured by the filmmakers. The sim center footage is masked to show only the monitor content, which is then overlaid on the filmmaker's camera angles. All video tracks are synchronized during import, ensuring consistent action across all angles. This setup allows for seamless cutting between camera angles while maintaining the correct monitor display throughout the edit. The synchronized tracks enable editors to switch between perspectives easily, knowing that the action and monitor content will remain consistent regardless of which angle is chosen (Figure 4).

**Figure 5:**
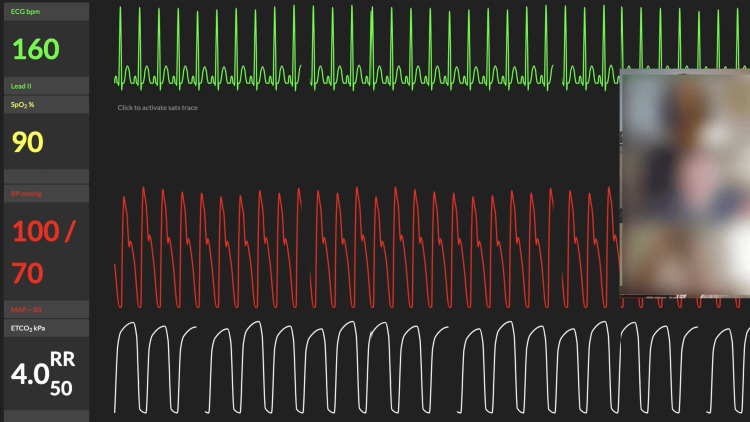
Remote simulation using ResusMonitor

If a dedicated simulation center is unavailable, a simpler alternative is to use a smartphone for video recording and incorporate free online vital sign monitors, such as ResusMonitor© [9]. Facilitators have two main options for integrating these tools into their simulations. One approach is to pre-record different vital sign states, such as initial, worsening, and improving conditions, and switch between these clips when learners request vital sign updates. Alternatively, facilitators can run ResusMonitor© in real time, screen-sharing live vital signs while toggling between the monitor and video content to provide a dynamic and interactive learning experience. Regardless of the chosen method, the goal is to enhance realism and increase engagement while keeping the workflow manageable for facilitators (Figure 5).

Enhancing Fidelity Through Immersive Video

**Figure 6:**
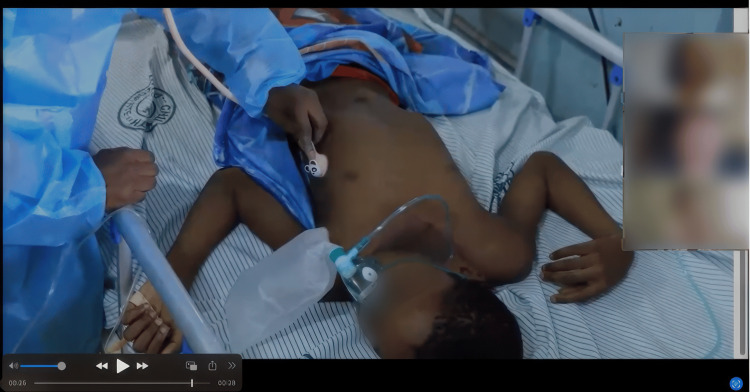
Remote simulation with Hotkeys, of a real Ghanaian patient Note: consent was obtained by the caregiver.

**Figure 7:**
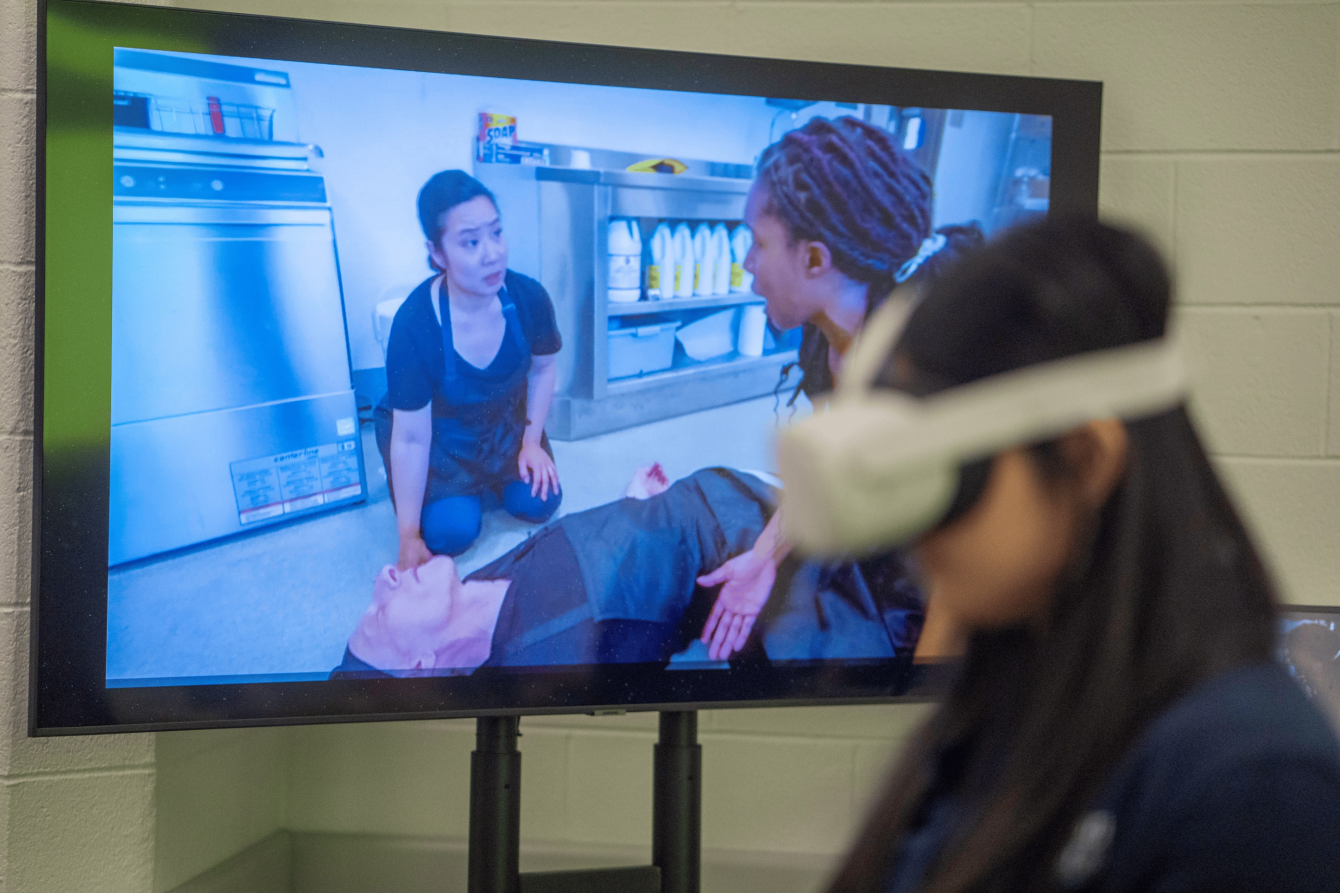
A student wearing a Meta Quest 3 Virtual Reality headset (Meta, California) watching a Virtual Reality (VR) front-end of an opioid overdose, mirrored on a classroom screen The individuals in this photo are actors in our VR front-end and have given permission for their image to be used.

A large part of Annenberg Hotkeys' functionality involves role-playing, with learners and facilitators taking on different roles to enhance engagement. Facilitators can heighten the sense of realism by incorporating various techniques. One approach is using real patient videos to provide authentic clinical scenarios (Figure 6). Another method is creating a contextual introduction video using a smartphone or an immersive 360° camera, allowing learners to experience the setting before the simulation begins (Figure 7). Additionally, facilitators can offer a virtual walk-through of the relevant healthcare environment, such as an emergency department, ambulance, or trauma bay, to further immerse participants in the scenario.

When learners engage with an immersive 360° video before the simulation, they can visualize the physical environment, reducing cognitive load and improving decision-making accuracy in time-sensitive situations. For example, if the facilitator is teaching new physicians how to respond to a trauma patient in the resuscitation bay in a particular healthcare setting, they can take a short (suggested <60 seconds) video walking through the resuscitation bay to orient the learner to the physical space; when learners watch this video during the simulation, they can visualize the physical space that helps suspend disbelief, or make the simulation feel more real. If the video is taken with immersive 360°, the students can watch the short video using a virtual reality head-mounted display, of which there are a variety ranging from dedicated units to inexpensive ones that use a simple smartphone as a screen. The introductory video may be the scene of an emergency department, automobile accident, the interior of an ambulance, a school lunchroom, or whatever meets the need of the facilitator. After watching the immersive video, the learner will remove his headset and participate in an Annenberg Hotkeys module role-playing that she is in this specific environment with those tools and resources. This allows one to re-use Annenberg Hotkeys modules for different scenarios and keep the experience fresh and interesting for the learner.

Assembling an Annenberg Hotkeys module from video clips

**Figure 8:**
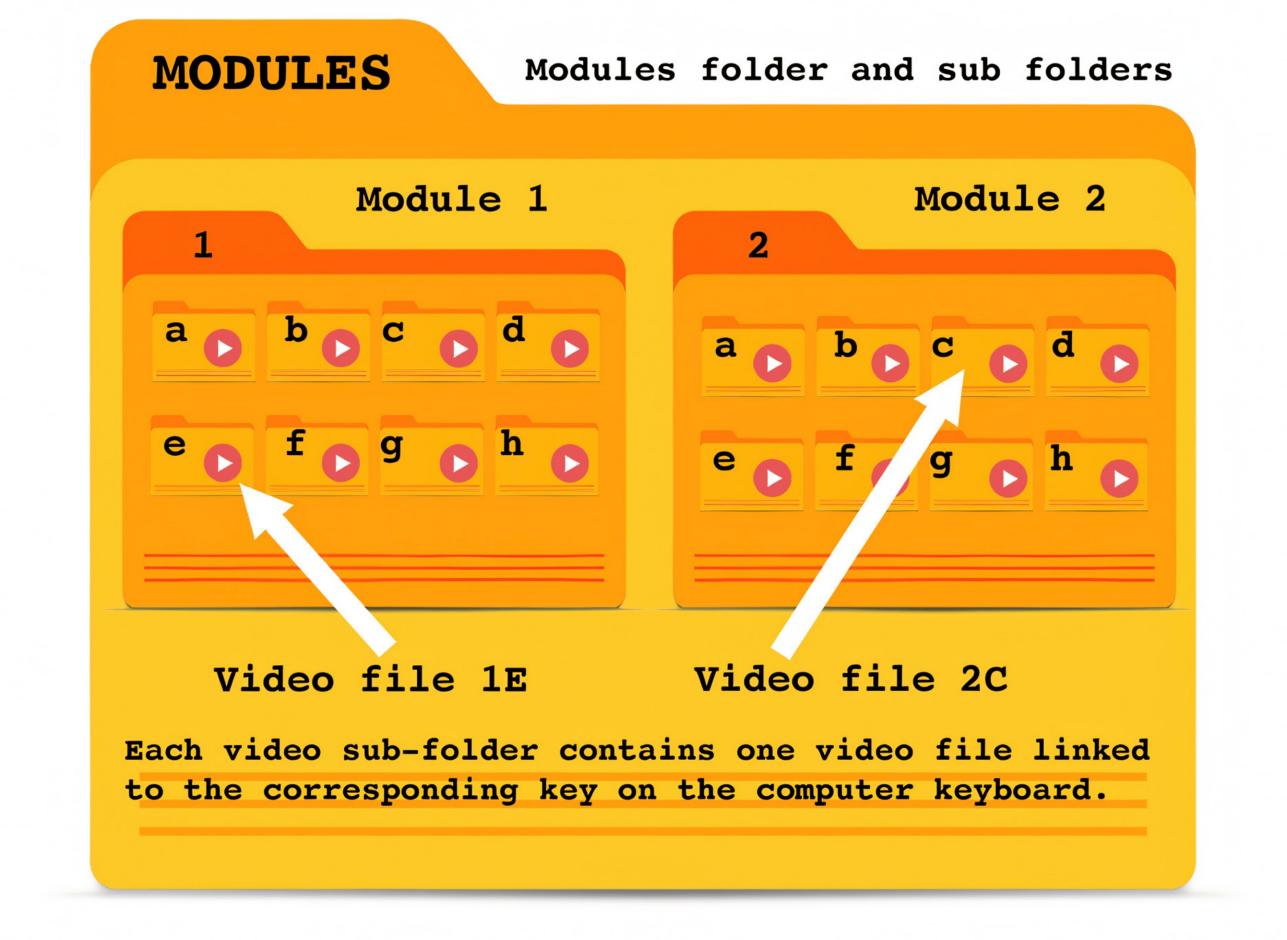
Hotkeys module structure

Multiple clips are assembled together into modules. Modules tell a story and each video represents a choice in that story made by the learners. Annenberg Hotkeys organizes and plays videos through a simple structured folder system. The system organizes content into numbered modules (0-9), each containing up to 26 lettered subfolders (a-z). Each subfolder can house a single .mp4 video file. When the application is running, users can quickly access specific videos using keyboard shortcuts. To play a video, users first press a number key (0-9) to select a module, followed by a letter key (a-z) to choose the specific video within that module. If staying within the same module, users can access different videos by simply pressing the corresponding letter key (Figure 8).

Keys can be mapped in any way the facilitator wishes, with similar videos next to one another on the keyboard or alphabetically. However the mapping is done, the facilitator should create a legend, study it, and refer to it during the simulation (Table 1). Another simple way to create the legend is to take a screenshot or picture with your phone of the folders expanded to show which video is linked to which key and module. As an example, for the case of pediatric shock, the videos in the below table are mapped to the keyboard keys for the facilitator to reference during the simulation. If the user types the letter “W” on their keyboard with the Annenberg Hotkeys software open, the video of the Basic Exam will immediately play without delay.

**Table 1:** Sample key mapping: legends of videos linked to computer keyboard keys NPA, nasopharyngeal airway; OPA, oropharyngeal airway; CPAP, continuous positive airway pressure; LMA, laryngeal mask airway; IO, intraosseous (referring to intraosseous access or infusion)

Key
A - Patient presented
Q - Monitors
W - Basic exam
R - Oxygen
T - Simple face mask
Y - Airway
U - Catheter suction
I - Yankauer other suction
O - NPA
P - OPA
S - Nasal cannula
D - CPAP
F - Ambu bag mask
G - Mapleson bag mask
H - Intubation
J - Intubation with LMA
K - IV access
L – Labs
Z - Labs with sugar
X - Antibiotics
C - Push-pull fluids
V - Fluids pump
B - IO electric
N - Recovery

Using a module

**Figure 9:**
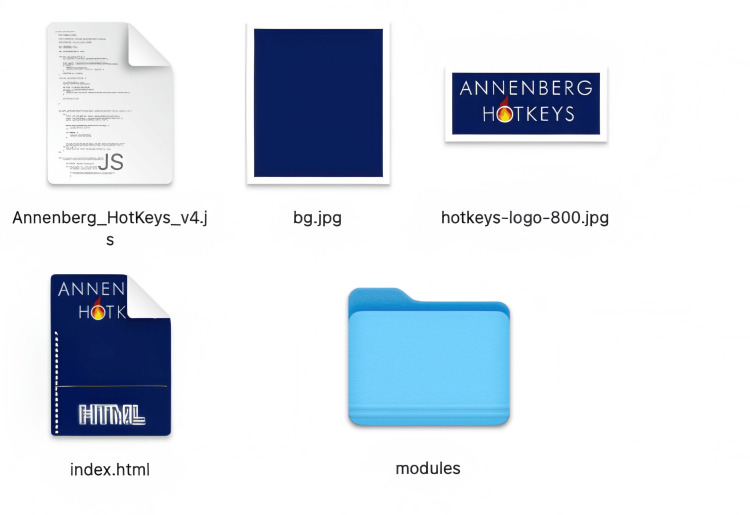
Screenshot of Annenberg Hotkeys zip file downloaded onto the computer

The simulation facilitator checks the Annenberg Hotkeys website [1] for up-to-date information about available modules and new developments. They then download the most up-to-date Annenberg Hotkeys zip file onto their computer and share the content via a web-based sharing program or directly via a thumb drive. Figure 9 shows how it appears on the computer.

**Figure 10:**
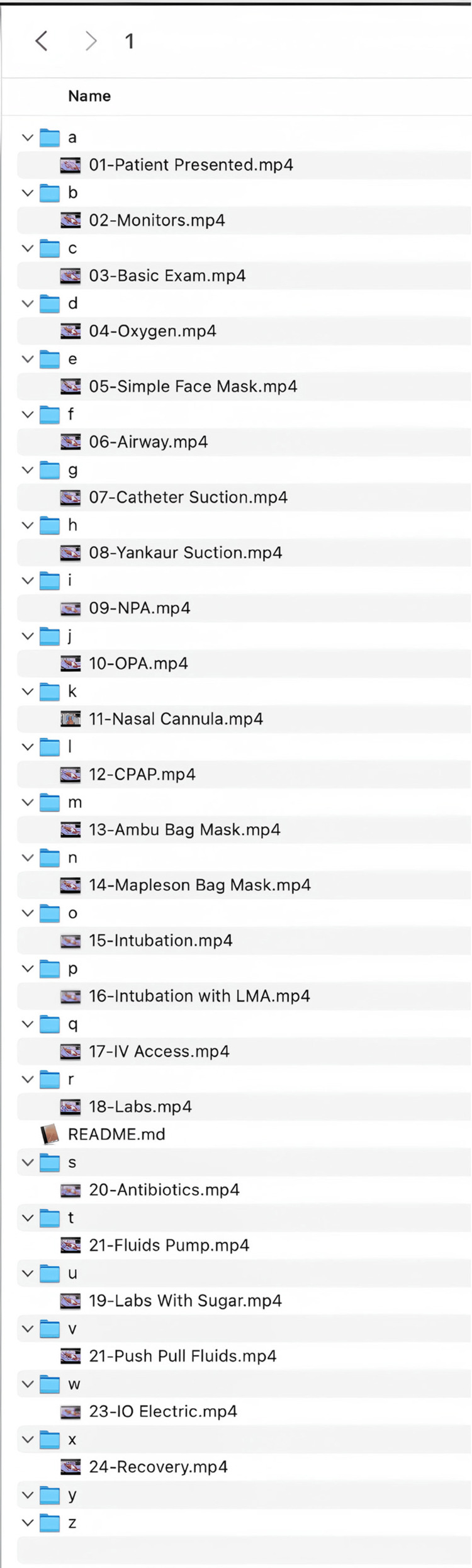
A screenshot showing how videos are placed inside module folders Facilitators can use a screen capture like this or create their own legend of key mapping to refer to while running the simulation.

Inside the modules folder, up to 10 sub-folders will either exist or can be created, numbered 0-9. Inside each of those module folders can be up to 26 mp4 video files in folders named a-z (Figure 10).

Annenberg Hotkeys can have up to 10 modules loaded at once. Each of these modules can have up to 26 videos. Since switching between modules is not appreciably more difficult than switching between videos, 260 video files are at your fingertips at the press of no more than two keys. Use numbers between 0-9 to select a module and then letters a-z for video files. There is no indication of module switching, so the learners are not distracted.

Annenberg Hotkeys will play any .mp4 video files stored in the correct place. This makes it simple to add custom content to an existing module for a procedure unique to your learning experience.

**Figure 11:**
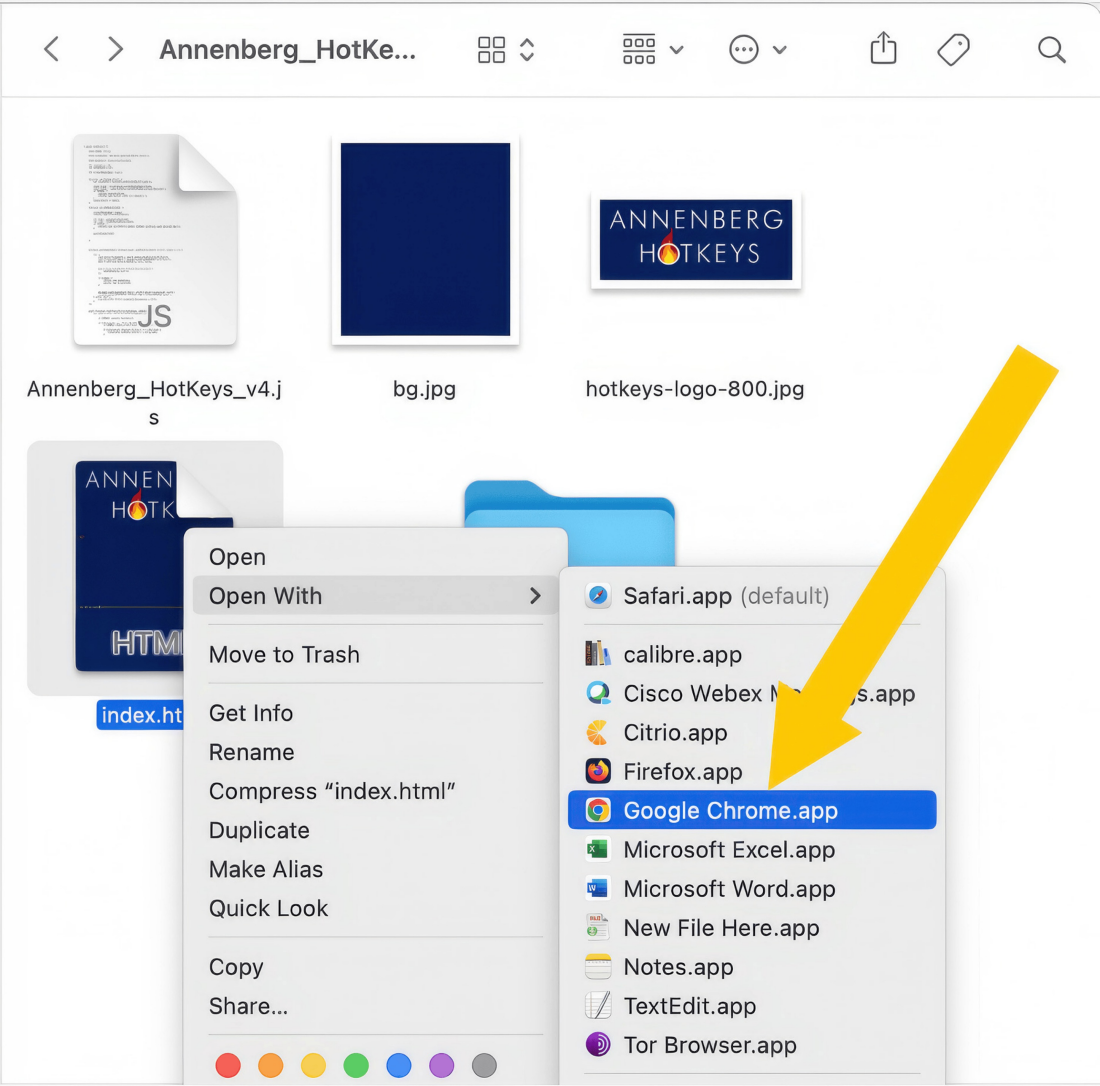
A screenshot demonstrating how to run Annenberg Hotkeys Right clicking on the index.html file and selecting “Open with” will allow one to select a browser that is not the default browser to launch Annenberg Hotkeys.

To start the simulation, the facilitator invites the participants to web conference meeting (i.e., Zoom©; Zoom Communications, Inc., California) and shares their screen. The facilitator then launches the index.html file (Figure 11) in one of the preferred browsers (as of this writing, Google Chrome©, Opera© or Microsoft Edge©). The splash screen will ask the facilitator to find the modules folder and allow the application to read it. By default, the modules folder is in the Hotkeys application folder, but there are good reasons why you may want to store modules on a network drive or even a flash drive. Once permission to read the contents of the modules folder is approved, the simulation can begin, and the facilitator can launch the first video. Video 1 shows a short video demonstrating this.

**Video 1:** Accessing Annenberg Hotkeys

The facilitator starts the simulation by hitting the module number (0-9) and then selecting the first video. In this simulation, the patient vignette is first presented to participants; then, the facilitator starts the first video. As the simulation progresses, participants request specific interventions or actions. The facilitator then consults their legend of available video responses, each corresponding to a specific keyboard key. When a participant requests an intervention, the facilitator presses the matching key on their computer keyboard, immediately playing the relevant video to advance the scenario. A second embedded facilitator playing a role such as parent, caregiver, witness, medic or nurse can be used to provide the learner with information when asked, such as the medical history, family history, or events leading up to event, and also steer learners away from distractions. For more information on how to do this, see the free and openly accessible Emergency SimBox resources on how to run a telesimulation drill [6].

For an audiovisual demonstration of Annenberg Hotkeys in use in a simulation demonstration, refer to Video 2.

**Video 2:** Hotkeys demonstration

Platform Evaluation and Performance Metrics

Annenberg Hotkeys has undergone multiple evaluation phases to assess its impact on medical training. Initial deployments at Penn Medicine and CHOP demonstrated high user satisfaction, with 90% of learners finding the platform intuitive and 85% reporting increased confidence in clinical decision-making. In its application for pediatric emergency medicine telesimulation in India and Ghana, evaluation methods included pre- and post-training assessments measuring improvements in diagnostic accuracy and intervention speed, instructor and learner surveys assessing usability and engagement, and process-of-care analyses demonstrating adherence to evidence-based protocols. These evaluation strategies align with best practices in simulation-based education, emphasizing the importance of learner-centered design and empirical assessment of training effectiveness [10,11].

Development Progression and Challenges Addressed

The development of Annenberg Hotkeys has evolved iteratively to address emerging challenges in medical simulation. In its initial version (2020), the platform was designed to support remote training during the COVID-19 pandemic. However, early adopters, particularly faculty, expressed discomfort with video-based simulation. To overcome this barrier, the design prioritized an intuitive user interface requiring minimal technical expertise. In its second iteration (2021), focused on pediatric emergency medicine training, a major challenge was the need for adaptive branching logic that could accommodate real-time learner decision-making. This was addressed by incorporating dynamic video pathways, enabling flexible simulation scenarios that responded to user input. The latest version (2022-present) has expanded to global implementation, presenting additional hurdles, such as internet connectivity limitations in low- and middle-income countries (LMICs). This was mitigated by introducing offline functionality, allowing educators and learners to access training materials without a continuous internet connection. Additionally, to enhance cultural relevance, the platform was adapted with region-specific patient cases and locally produced videos, ensuring that learners could engage with content contextualized to their healthcare environments. These iterative refinements reflect principles of human-centered design and adaptive learning strategies, which have been shown to enhance the effectiveness of simulation-based medical education [12,13].

Grounding in Simulation-Based Learning Theory

The theoretical foundation of Annenberg Hotkeys aligns with established principles of simulation-based learning and instructional design. Kolb’s Experiential Learning Theory underscores the importance of active engagement in learning, which is a core feature of Annenberg Hotkeys' interactive decision-making structure [14]. Additionally, Miller’s Pyramid of Clinical Competence provides a framework for understanding how the platform facilitates a progression from knowledge acquisition (“knows”) to skill demonstration (“does”) through immersive simulation experiences [15]. The platform's user interface and content delivery are further informed by cognitive load theory [16], which guided the design to prevent cognitive overload and ensure that learners can effectively process information in high-stress scenarios. By integrating these theoretical frameworks, Annenberg Hotkeys enhances clinical training outcomes and supports learners in developing actionable skills applicable to real-world patient care.

## Discussion

Annenberg Hotkeys was developed through an interdisciplinary collaboration between filmmakers, software designers, and physicians at the University of Pennsylvania and the Children’s Hospital of Philadelphia. This partnership aimed to address key challenges in synchronous simulation-based education, particularly when facilitators and learners are geographically separated or face resource limitations. It can be used by educators who want to access pre-made videos, available on the website [1], of clinical scenarios or skills demonstrations, or the tool can be utilized by those who want to create their own content inputted into the free software. Annenberg Hotkeys is designed to integrate with either facilitator newly created guides or with other free and openly accessible simulation facilitator guides [6,7]. Educators with little or even no background in video editing or using technology to facilitate hybrid (remote and in person) [4] simulation-based education modules have demonstrated being able to not just use this technique but also create their own content and train others, in their unique healthcare settings, on how to do the same. It has been used with success in both resource-rich and resource-limited settings. Applications for its use are boundless, from medical education and beyond. Existing videos and the most recent version of the Annenberg Hotkeys software can be found on the website [1].

Limitations

Through the development and implementation of Annenberg Hotkeys, we identified several technical and instructional challenges that educators should consider when adopting the platform. First, video playback performance depends on the capabilities of the host machine, and large file sizes may impact usability, particularly in settings with limited bandwidth. To optimize performance, we recommend limiting video resolution to 720p (standard HD) to reduce download times and ensure smoother playback over slower internet connections.

Additionally, when facilitating a simulation, educators should carefully consider video length. Shorter videos may require frequent repetition or pausing for discussion, while longer videos may need strategic segmenting to maintain participant engagement. If using the ResusMonitor© website concurrently, facilitators can switch back to the vital sign monitor between video interventions, allowing participants to request the next hotkey-triggered clip as needed. Alternatively, incorporating an ever-ready cutaway video, such as a close-up of a nurse or team performing a task, can help sustain visual engagement during pauses or extended discussions.

While our observations suggest that Annenberg Hotkeys enhances simulation-based learning, we acknowledge that this report lacks quantitative data on learner outcomes or the platform’s overall effectiveness. Future studies should include structured assessments to measure its impact on participant performance and engagement. Additionally, although we developed VR front-end modules as part of the platform, we have not yet collected performance data or user feedback on this feature. Further evaluation is needed to determine the usability and educational value of these components.

Future directions

While Annenberg Hotkeys was initially used to train healthcare workers and medical students during the COVID-19 pandemic, its flexible design allows for applications across a wide range of educational and professional contexts. Beyond medical training, the platform has the potential to enhance learning in diverse disciplines and serve as a research tool for investigating educational, psychological, and behavioral outcomes.

For instance, could different approaches to presenting information influence decision-making? Would teenagers make different choices about risk behavior if scenarios were introduced in varied ways? Could political leaders alter their policy decisions if they witnessed the real-world consequences of policy enactment? Might consumers rethink their resource allocation or conservation efforts if they saw the direct impact on biodiversity, climate, and human communities? Similarly, how would business leaders navigate complex ethical dilemmas if they could observe the cascading effects of their choices on stakeholders, corporate reputation, and long-term business sustainability?

Future studies may also explore the role of technology in shaping learning outcomes, particularly through the use of immersive media such as 360° video and VR. We have already developed optional VR front-end modules that immerse learners in high-fidelity environments as a preparatory step before transitioning to flat-screen lessons. However, we have yet to formally evaluate whether this added immersion enhances perceived learning or improves comprehension of key instructional objectives.

There are many exciting opportunities to expand Annenberg Hotkeys. Future enhancements could include conceptual initiatives, such as workshops with diverse user groups to explore both medical and non-medical applications, as well as logistical improvements like developing a centralized hosting system for storing and distributing user-created modules. Additionally, pedagogical advancements, such as best-practice guides for designing and implementing user-generated content, could further refine its educational impact.

At its core, Annenberg Hotkeys is a simple yet powerful tool that harnesses human creativity, curiosity, and the innate desire to engage in play. By offering an alternative to expensive, high-fidelity simulation experiences, it provides an accessible, adaptable, and widely available approach to experiential learning.

## Conclusions

Annenberg Hotkeys was developed to promote education by providing free, accessible tools for diverse learning environments. This technical report offers a step-by-step guide to creating an Annenberg Hotkeys module, covering scenario development, video production, and monitor integration. Educators can incorporate either pre-made videos or their own original content into a synchronous learning module, enabling flexible and interactive simulation-based instruction.

The report also provides practical tips for video creation, editing, and scenario design, helping users develop customized training experiences. Additionally, it outlines specific ways to use Annenberg Hotkeys for simulation-based medical education, whether in-person or remote, by integrating pre-made videos, open-source facilitator guides, and monitor simulators. The Annenberg Hotkeys team is committed to expanding the global reach of free, web-based medical education and welcomes partnerships and feedback for further applications of the tool.
